# Supplementary material for: Autocatalytic amplification of Alzheimer-associated Aβ42 peptide aggregation in human cerebrospinal fluid
Source: Commun Biol. 2019 Oct 8;2:365. doi: 10.1038/s42003-019-0612-2 (PMC6783456; doi:10.1038/s42003-019-0612-2)
Supplement: Supplementary file 1 — Supplementary information [file 42003_2019_612_MOESM1_ESM.pdf]

Supplementary Figures

Validation of automated pipetting

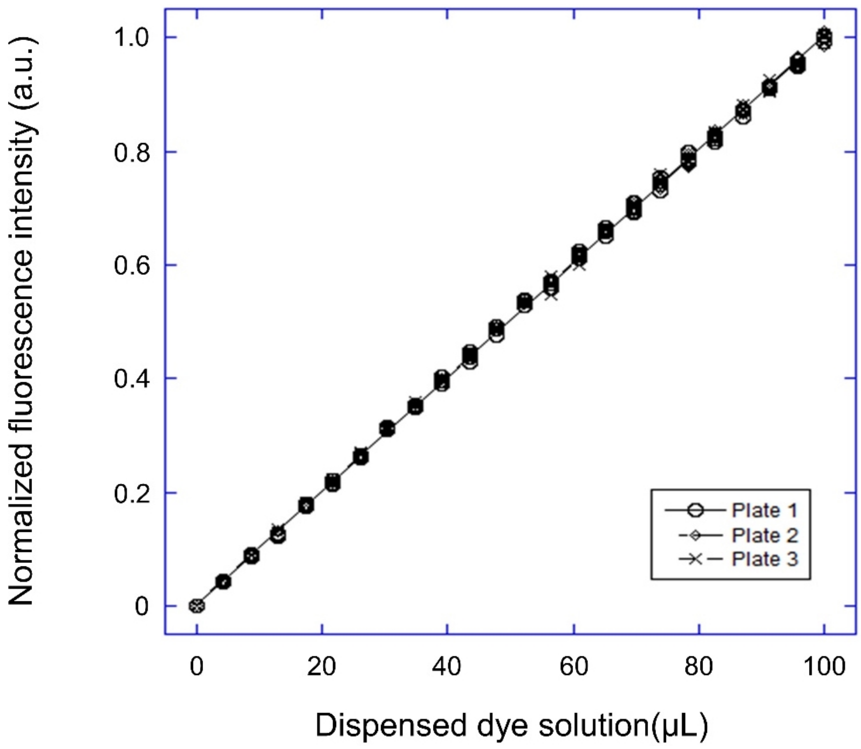

Supplementary Fig. 1. Validation of automated pipetting. The fluorescence intensity profile of three independent pyranine dilution series created by automated pipetting. Channel A was loaded with 2 µM pyranine dye dissolved in 50 mM bicine pH 9.0 and channel B with 50 mM bicine pH 9.0. The gradient was created in a 96-well plate by gradually varying the output from the channels, from 100 µl A/0 µl B to 0 µl A/100 µl B. The fluorescence was recorded in a CLARIOstar plate reader (BMG Labtech). The three series were performed with 24 different concentrations, each concentration done in quadruplicates in each series. The results show a high degree of linearity between expected concentration and fluorescence intensity as well as high reproducibility both within and between the independent repeats (R value < 0.999 for all three series).

Additional Aβ42 aggregation kinetics data in buffer

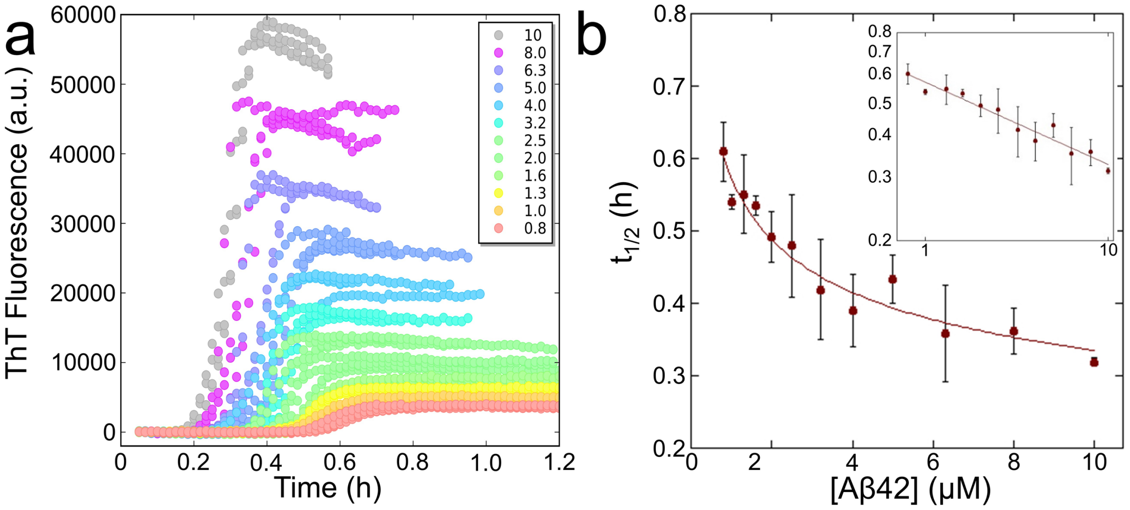

Supplementary Fig. 2. Aggregation kinetics in buffer. (a) The ThT fluorescence as a function of time (h) for a range of Aβ42 monomer concentrations, 0.8-10 μM, studied in 20 mM Hepes/NaOH, 140 mM NaCl, 1 mM $CaCl_2$, pH 8.0 with 10 μM ThT. The aggregation curves from another experiment are shown in Fig. 1a. (b) The extracted $t_{1/2}$, fitted with eq. 1 as described in the main text. The error bars are the standard deviation of the replicates, where n=4 for all concentrations except 1.3 μM where one outlier was removed.

Effect of CSF concentration

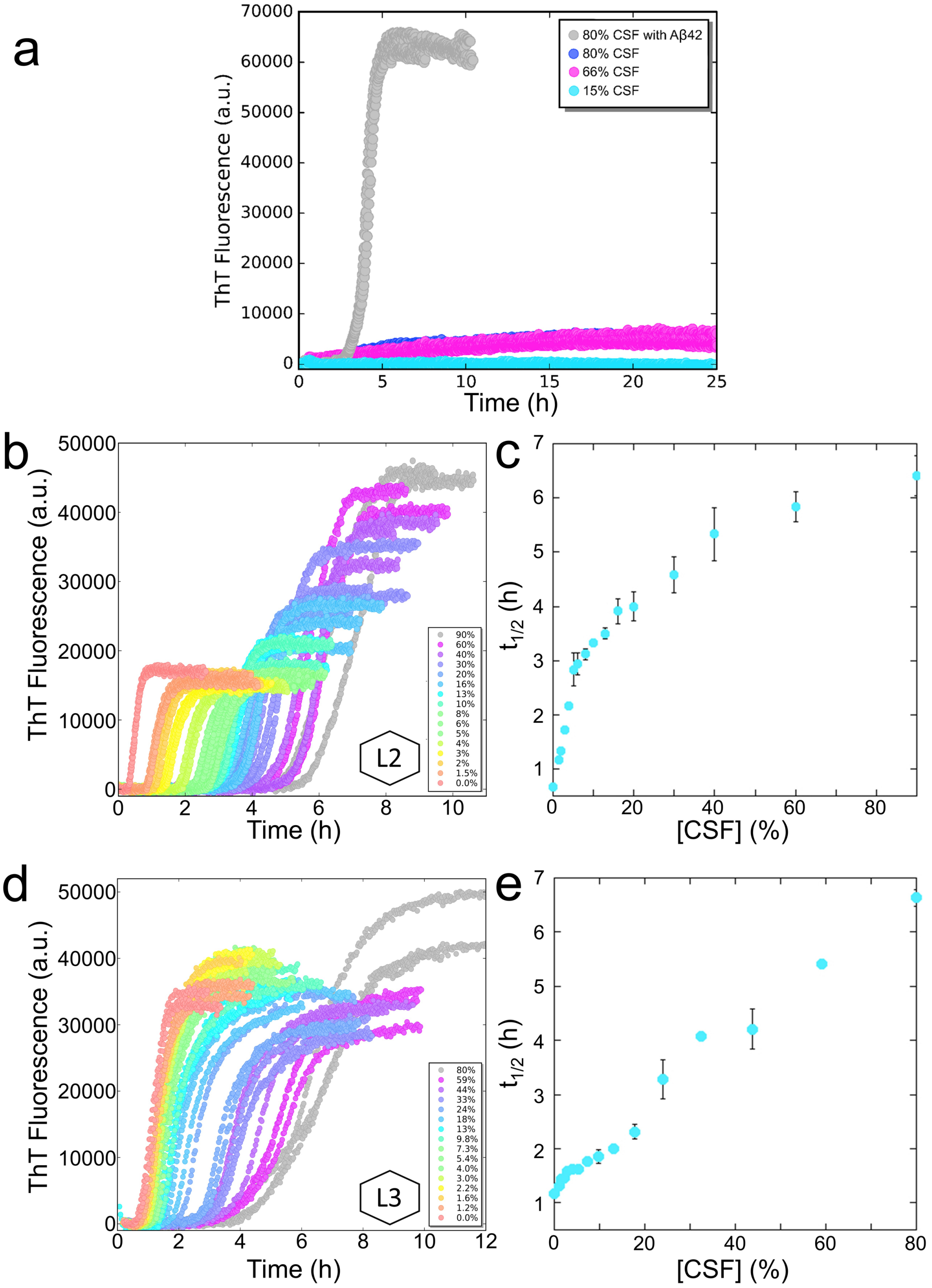

Supplementary Fig. 3. Aggregation kinetics in CSF. The ThT fluorescence as a function of time (h) for (a) 0 or (b) and (d) 3 μM Aβ42 in various concentrations of CSF (0-80%) in 20 mM Hepes/NaOH, 140 mM NaCl, 1 mM $CaCl_2$, pH 8.0 with 10 μM ThT. The aggregation curves from another experiment are shown in Fig. 2a. (c) and (e) are the extracted $t_{1/2}$ plotted against the CSF concentration, from (b) and (d) respectively. The error bars are the standard deviation of the replicates, where n=3. The hexagonal symbol represents the CSF pool used.

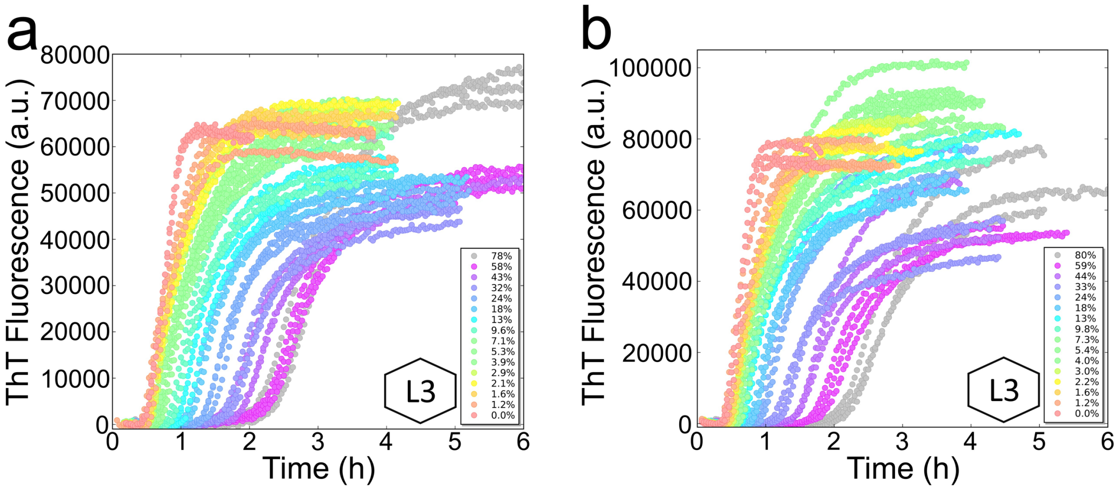

Supplementary Fig. 4. Aggregation kinetics in constant Aβ42 concentration. The ThT fluorescence as a function of time (h) for 5 µM Aβ42 in various concentrations of CSF (0-80%) in 20 mM Hepes/NaOH, 140 mM NaCl, 1 mM $CaCl_2$, pH 8.0 with 10 µM ThT. The hexagonal symbol represents the CSF pool used. (a) and (b) are two repeats of the experiment.

A slight difference in maximum ThT intensity can be seen for the different runs, and is summarized in Supplementary Fig. 5. What this is due to is at this point unknown; however, as it does not seem to affect the kinetics, it was not investigated further in this paper.

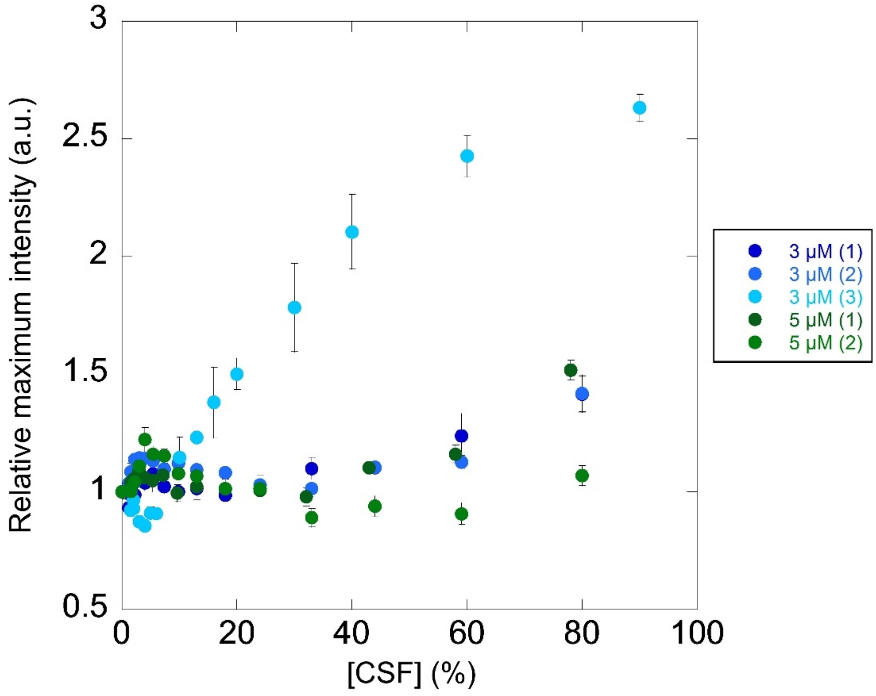

Supplementary Fig. 5. Maximum ThT intensity. Normalized maximum ThT intensity relative 0 %CSF as a function of CSF concentration, for samples containing 3 µM (blue) or 5 µM (green) Aβ42. The error bars are the standard deviation of the replicates, where n=3. The series 3 µM (2) and 5 µM (1) + (2) are made with the same CSF pool, while 3 µM (1) is made with another and 3 µM (3) is made with a third.

Aβ42 aggregation kinetics at constant CSF concentration

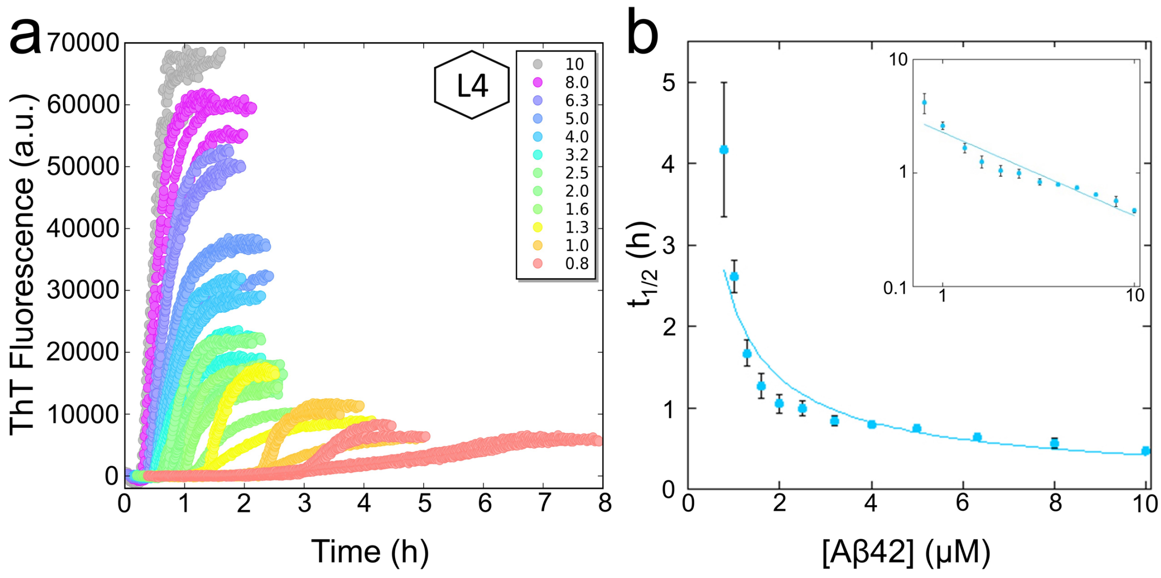

Supplementary Fig. 6. Aggregation kinetics in 15% CSF. (a) The ThT fluorescence as a function of time (h) 0.8-10 μM Aβ42 in 15% CSF and 20 mM Hepes/NaOH, 140 mM NaCl, 1 mM $CaCl_2$, pH 8.0 with 10 μM ThT. The hexagonal symbol represents the CSF pool used. (b) The extracted $t_{1/2}$, fitted with eq. 1 as described in the main text. The error bars are the standard deviation of the replicates, where n=4. The aggregation curves from another experiment are shown in Fig. 4a.

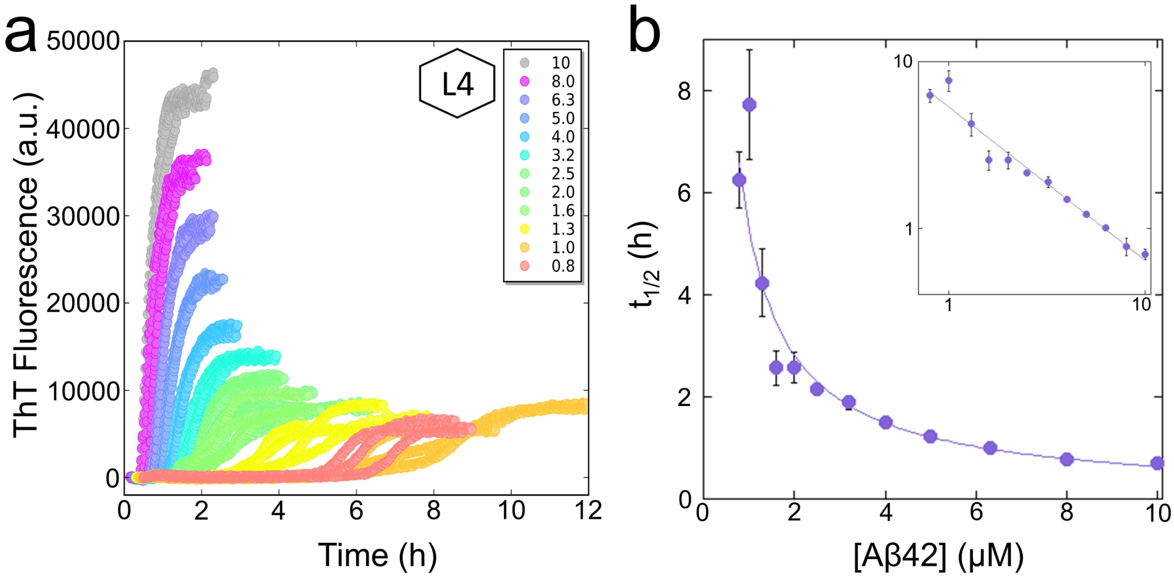

Supplementary Fig. 7. Aggregation kinetics in 32% CSF. (a) The ThT fluorescence as a function of time (h) 0.8-10 μM Aβ42 in 32% CSF and 20 mM Hepes/NaOH, 140 mM NaCl, 1 mM $CaCl_2$, pH 8.0 with 10 μM ThT. The hexagonal symbol represents the CSF pool used. (b) The extracted $t_{1/2}$, fitted with eq. 1 as described in the main text. The error bars are the standard deviation of the replicates, where n=4 for all concentrations except 0.8 μM where one outlier was removed. The aggregation curves from another experiment are shown in Fig. 4b.

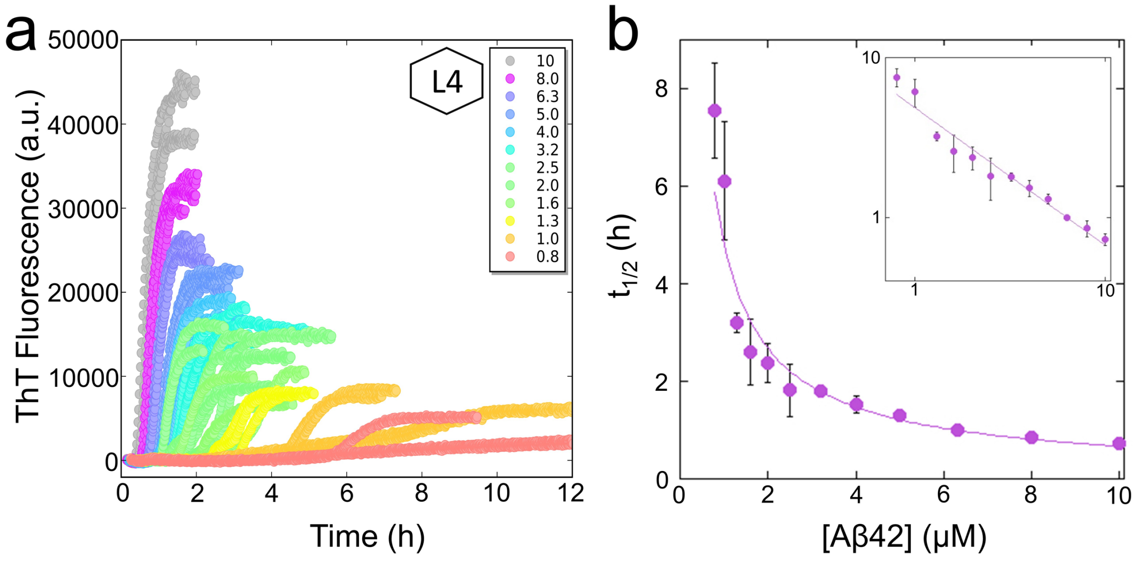

Supplementary Fig. 8. Aggregation kinetics in 66% CSF. (a) The ThT fluorescence as a function of time (h) 0.8-10 μM Aβ42 in 66% CSF and 20 mM Hepes/NaOH, 140 mM NaCl, 1 mM $CaCl_2$, pH 8.0 with 10 μM ThT. The hexagonal symbol represents the CSF pool used. (b) The extracted $t_{1/2}$, fitted with eq. 1 as described in the main text. The error bars are the standard deviation of the replicates, where n=4 for all concentrations except 1.3 μM and 0.8 μM where one outlier per concentration was removed. The aggregation curves from another experiment are shown in Fig. 4c.

Kinetic analysis

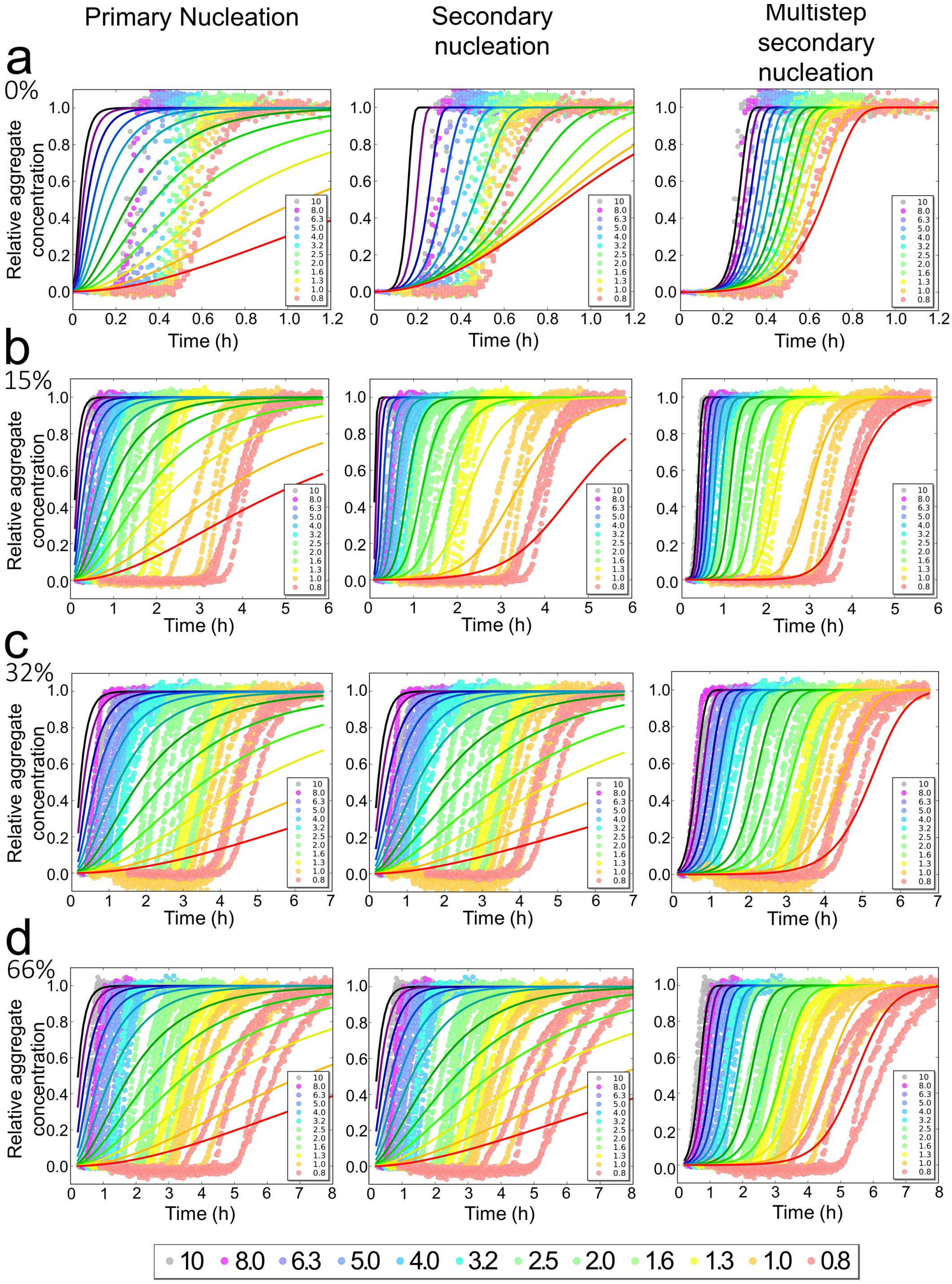

Supplementary Fig. 9. Normalized kinetics with fitted models. The data with fits created using AmyloFit, with normalized ThT fluorescence (relative aggregate concentration) as a function of time (h) for 0.8-10 μM Aβ42 in buffer with (a) 0% CSF, (b) 15% CSF, (c) 32% CSF, and (d) 66% CSF. The left graphs are fitted primary nucleation and elongation; the middle graphs are fitted with non-saturated secondary nucleation dominated; the graphs in the right column are fitted with multistep secondary nucleation model. The graphs in the left and the right columns are also shown in Fig. 6. The legend – with the Aβ42 concentrations in μM – at the bottom is the same as in the plots, only magnified for clarification.

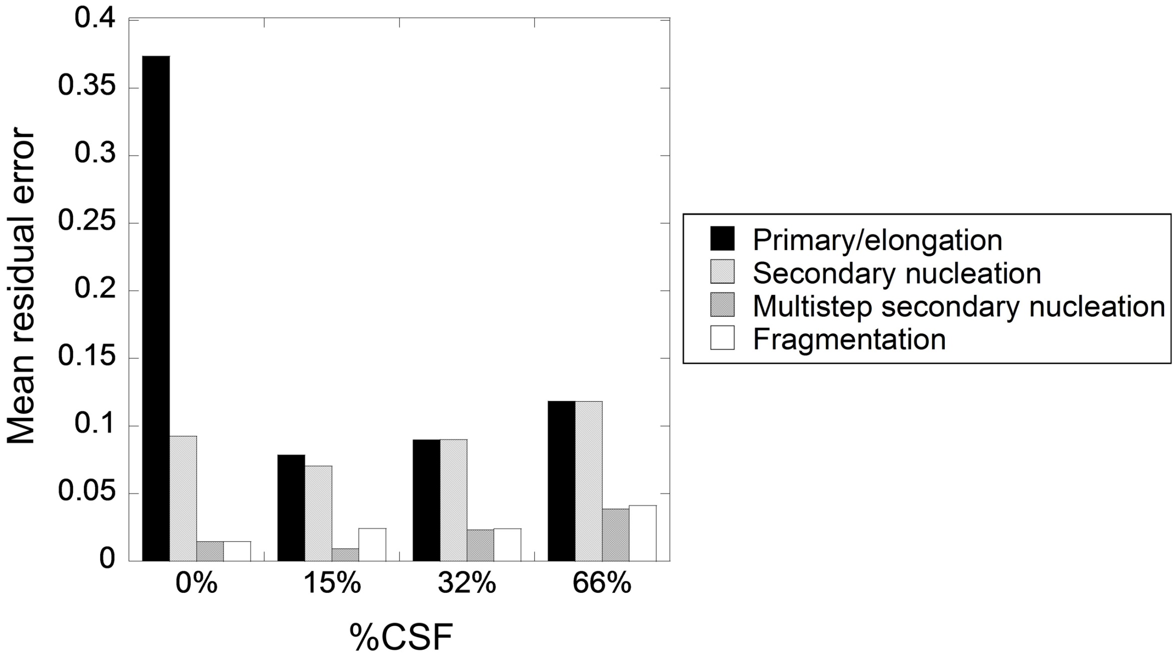

Supplementary Fig. 10. **Mean residual errors.** The mean residual error for the fits of four different models created using AmyloFit, for 0.8-10 μM Aβ42 in buffer and 0% CSF (over 8680 datapoints), 15% CSF (over 24188 datapoints), 32% CSF (over 36988 datapoints) and 66% CSF (over 36096 datapoints) (Fig. 6 and Supplementary Fig. 9). Black: primary nucleation and elongation. Striped: primary nucleation, secondary nucleation and elongation. Hatched: primary nucleation, multistep secondary nucleation and elongation. Open: primary nucleation, fragmentation and elongation.

Seeding experiments

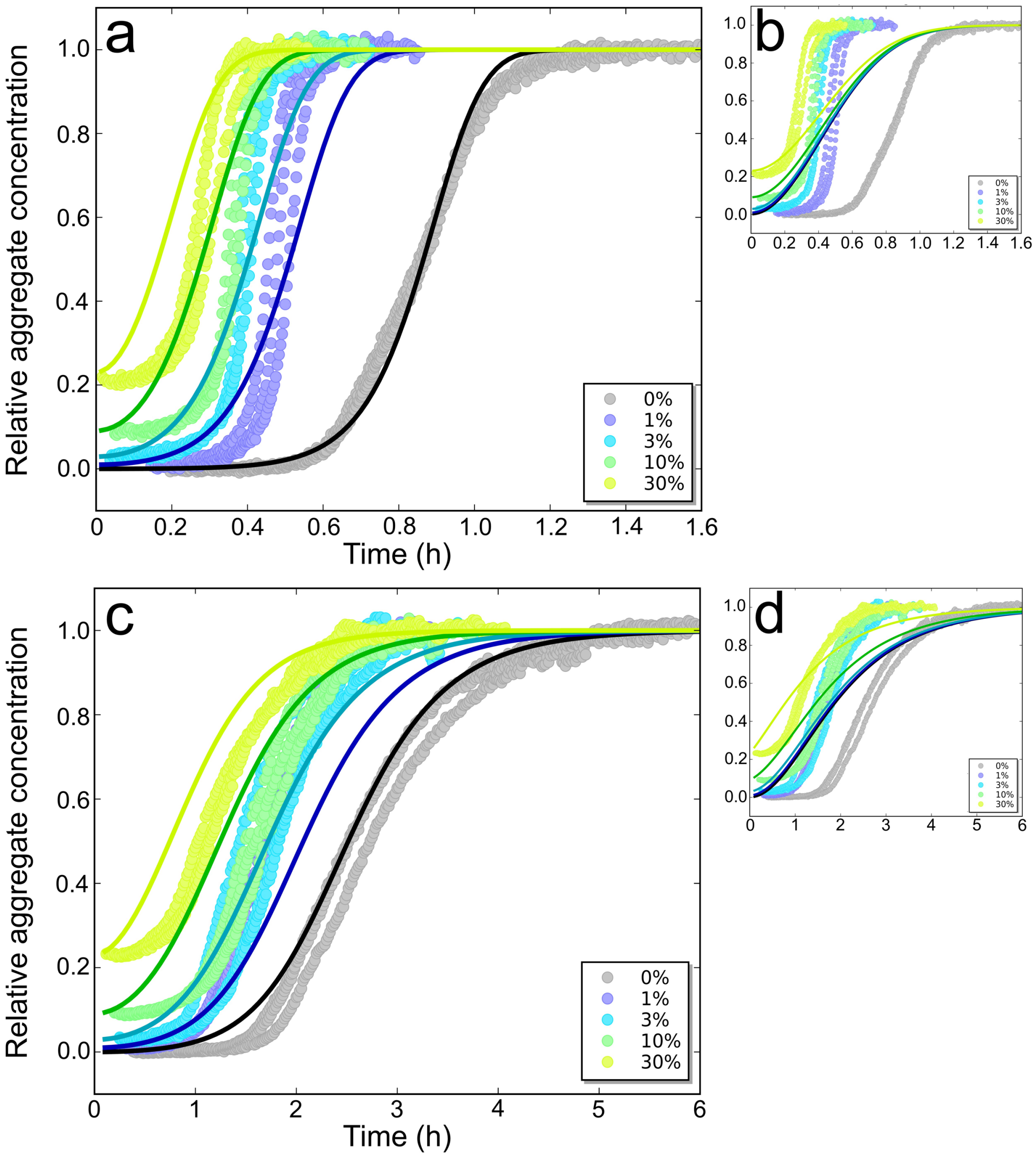

Supplementary Fig. 11. Seeded data. Data from the seeded experiments with 3 μM Aβ42 monomer concentration in 20 mM Hepes/NaOH, 140 mM NaCl, 1 mM $CaCl_2$, pH 8.0 with 10 μM ThT and addition of 0-30% seeds in (a-b) the absence of CSF and in (c-d) the presence of 66% CSF with fits using a multi-step secondary nucleation model (a, c), and primary nucleation and elongation (b, d).

Supplementary Tables

Characterization of the CSF pools

Supplementary Table 1. Comparison of two CSF pools. Characteristics of the two CSF pools used in the kinetic experiments shown in Fig. 1, 4 and Supplementary Fig. 6-8. The pH was found to be similar (Lund pool) or higher (Gothenburg pool) than the buffer (pH 8.0). The conductivities were somewhat lower than that of the buffer (12 mS). Protein content was measured using the Bradford assay with human IgG as a protein standard.

	Lund (L4)	Gothenburg (G1)
pH	8.0	8.7
Conductivity	9 mS	8 mS
Protein content	0.55 mg·ml^{-1}	0.36 mg·ml^{-1}

Kinetic analysis

Supplementary Table 2. **Parameters.** The fitted parameters for the multistep secondary nucleation model used in Fig. 6.

	**0%**	**15%**	**32%**	**66%**
#datapoints	8680	24188	36988	36096
Mean residual error	0.014554	0.00914	0.023154	0.038671
k_+k_n (M^{-nc} h^{-2})	0.158154	6.89E+13	6.70E+14	4.86E+14
n_c	0.00001	3	3	3
k_+k_2 ($M^{-1}h^{-2}$)	2.08E+24	1.29E+19	2.11E+21	4.17E+19
n_2	2	2	2	2
K_M (M^2)	2.30E-17	2.14E-12	8.55E-16	4.68E-14
