# Supplementary material for: Autocatalytic amplification of Alzheimer-associated Aβ42 peptide aggregation in human cerebrospinal fluid
Source: Commun Biol. 2019 Oct 8;2:365. doi: 10.1038/s42003-019-0612-2 (PMC6783456; doi:10.1038/s42003-019-0612-2)
Supplement: Supplementary file 2 — Description of additional supplementary data [file 42003_2019_612_MOESM2_ESM.docx]

**Supplementary data 1**

Shows the data used to create Figure 1.

**Tab 1A**

The first column contains the time x-axis in units of hours. The following columns contains the raw fluorescence intensity values for each concentration as a function of time.

**Tab 1B**

The first column contains all Aβ concentrations in µM. The second column lists all the calculated half times in hours. The third column lists the mean half times of each concentration and the fourth column lists the associated standard deviations.

**Supplementary data 2**

Shows the data used to create Figure 2.

**Tab 2A**

The first column contains the time x-axis in units of hours. The following columns contains the raw fluorescence intensity values for each concentration as a function of time.

**Tab 2B**

The first column contains all CSF concentrations in percent. The second column lists all the calculated half times in hours. The third column lists the mean half times of each concentration and the fourth column lists the associated standard deviations.

**Supplementary data 3**

Shows the data used to create Figure 4.

**Tab 4A-4C**

The first column contains the time x-axis in units of hours. The following columns contains the raw fluorescence intensity values for each concentration as a function of time.

**Tab 4D-E**

The first column contains all Aβ concentrations in µM. The second column lists all the calculated half times in hours. The third column lists the mean half times of each concentration and the fourth column lists the associated standard deviations. This is repeated for all CSF concentrations in order (0%, 15%, 32% and 66%)

**Supplementary data 4**

Shows the data used to create Figure 6.

The ‘data’ tabs contain the normalized data that were used to fit the models. For each concentration the Y-values (normalized fluorescence intensity) are listed with their corresponding X-values (time in hours).

The ‘Fit’ tabs contain the best fit with a multistep secondary nucleation model evaluated for each concentration at each of the X-values.

The ‘inset’ tabs contain the best fit with a primary nucleation and elongation model evaluated for each concentration at each of the X-values.

**Supplementary data 5**

Shows the data used to create Figure 7.

**Tab 7A, 7C**

The first column contains the time x-axis in units of hours. The following columns contains the raw fluorescence intensity values for each concentration as a function of time.

**Tab 7B, 7D**

The first column contains all seed concentrations in %. The second column lists all the calculated half times in hours. The third column lists the mean half times of each concentration and the fourth column lists the associated standard deviations.
